# Photon acceleration and tunable broadband harmonics generation in nonlinear time-dependent metasurfaces

**DOI:** 10.1038/s41467-019-09313-8

**Published:** 2019-03-22

**Authors:** Maxim R. Shcherbakov, Kevin Werner, Zhiyuan Fan, Noah Talisa, Enam Chowdhury, Gennady Shvets

**Affiliations:** 1 School of Applied and Engineering Physics, Cornell University, Ithaca, NY 14853 USA; 2 Department of Physics, The Ohio State University, Columbus, OH 43210 USA

## Abstract

Time-dependent nonlinear media, such as rapidly generated plasmas produced via laser ionization of gases, can increase the energy of individual laser photons and generate tunable high-order harmonic pulses. This phenomenon, known as photon acceleration, has traditionally required extreme-intensity laser pulses and macroscopic propagation lengths. Here, we report on a novel nonlinear material—an ultrathin semiconductor metasurface—that exhibits efficient photon acceleration at low intensities. We observe a signature nonlinear manifestation of photon acceleration: third-harmonic generation of near-infrared photons with tunable frequencies reaching up to ≈3.1*ω*. A simple time-dependent coupled-mode theory, found to be in good agreement with experimental results, is utilized to predict a new path towards nonlinear radiation sources that combine resonant upconversion with broadband operation.

## Abstract (web summary)

Photon acceleration, which can be used to generate tunable high harmonic radiation, typically requires high-intensity lasers and long propagation distances. Here, Shcherbakov et al. show efficient photon acceleration at low power input power from a semiconductor metasurface, less than a micron thin.

## Introduction

Before the demonstration of the first laser by Theodore Maiman, light propagation was widely considered to be a linear process, with the photons not expected to interact with each other. This simple understanding of light−matter interactions was overturned in the early 1960s in second harmonic generation experiments by Franken et al.^1^. From this demonstration of the merger between two photons into a photon with doubled energy, the nonlinear optics was born. Subsequent realizations of the third^2^ and higher-order^3^ harmonics enabled efficient light sources^4^, high-resolution microscopy^5,6^, and produced some of the most sensitive optical characterization techniques^7–9^.

However, fundamental effects limit the efficiency and spectral range of the canonical nonlinear processes. Mainly, the very nature of the standard *n*-photon processes (where *n* ≥ 2 is an integer) dictates that a narrow-band laser pulse centered at the frequency *ω*_L_ with the spectral width Δ*ω*_L_ cannot produce upconverted photons with the frequencies Ω_*n*_ outside of the $|{\mathrm{\Omega }}_n - n\omega _{\mathrm{L}}| \lesssim \sqrt n \Delta \omega _{\mathrm{L}}$ spectral interval. While using high-finesse optical cavities or other resonant structures can enhance the efficiency of harmonics generation, they even further limit the spectral range of the nonlinearly generated photons. Thus, a fundamental challenge is to find a nonlinear optical process that enables efficient nonlinear frequency conversion without sacrificing the spectral bandwidth. In this article, we propose and experimentally realize one such process: upconversion of mid-infrared (MIR) light undergoing rapid blue-shifting—also known as photon acceleration^10^—in a resonant nonlinear metasurface.

The concept of photon acceleration (PA) was originally introduced in gaseous plasmas^11,12^ as a process of frequency conversion that occurs when electromagnetic waves propagate in a medium with a time-dependent refractive index^13^. A reduction of the refractive index via free carrier (FC) generation results in a measurable blue-shifting regardless of whether the FCs were produced by the radiation itself^11,14^ or by an auxiliary electromagnetic pulse^15^, as well as in the broadening of the spectrum^16^, which was demonstrated for harmonics generation as well^17^. PA in a solid (e.g., semiconductor) medium can be achieved at much lower laser intensities than in a gas because of the ease of FC generation^18,19^, and can be further enhanced in high-quality factor (high-*Q*) optical cavities. For example, loading photons into a ring microcavity^20^ or a photonic crystal cavity^21^ and subsequently generating FCs by an external pump while the photons are still in the cavity resulted in continuous near-infrared wavelength shifts of up to several nanometers^20,22^. However, no nonlinear manifestations were observed, primarily because of high sensitivity of high-*Q* resonators to high-intensity near-infrared light. Therefore, new photon-accelerating platforms based on free-space light coupling are needed.

Recently, a new paradigm of regularly nanostructured surfaces—metasurfaces^23–25^—has been established for ultrathin nonlinear and active materials^26,27^. While metasurfaces share with optical cavities the attractive properties of high spectral selectivity and strong field concentration, their important feature is the strong coupling to free-space beams. A variety of metasurface designs have been implemented for applications as diverse as wave front manipulation (in both linear^28^ and nonlinear^29^ regimes), rapid amplitude and phase modulation^30–33^, as well as efficient harmonics generation^34–36^ and all-optical modulation^37–39^. Of particular interest are semiconductor-based metasurfaces that utilize strong, geometry-dependent Mie-type localized modes^40^ with high-*Q*-factors^41,42^. They have already shown record-breaking nonlinear optical performance on the nanoscale^43–46^, making them an attractive platform for observing PA and tunable and broadband optical harmonics.

**Fig. 1:**
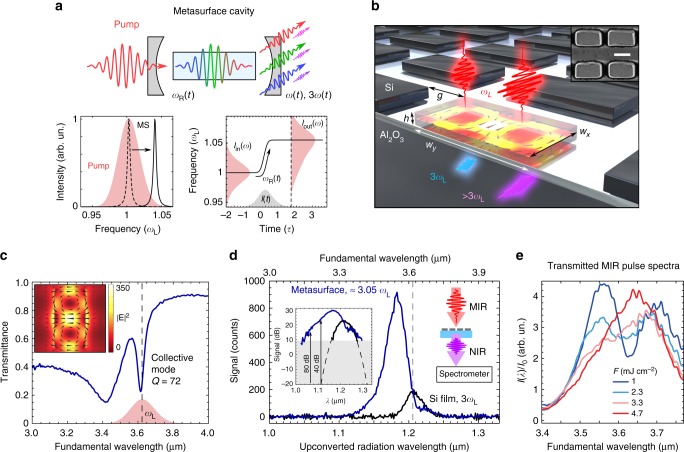
Self-induced blue-shift of harmonics from a nonlinear photon accelerating semiconductor infrared metasurface (PASIM). **a** The concept of blue-shifted harmonics generation. Mid-infrared (MIR) photons are trapped by the metasurface (MS) cavity, blue-shifted by the rapid refractive index variation due to free carrier generation inside the MS, and then nonlinearly upconverted to near-infrared (NIR) photons via the standard THG process. The blue-shifted MIR and NIR photons then leave the MS, and their spectra are detected in transmission. Red shaded areas in the plots illustrate the radiation spectra, the gray area represents the temporal profile of the incoming pulse. Solid and dashed curves illustrate the time-dependent nature of the MS resonance. **b** Schematic of the sample and the MIR beam setup. In experiments, the following dimensions of the sample were used: *w*_*x*_ = 0.87 μm, *w*_*y*_ = 1.54 μm, *g* = 400 nm, and *h* = 600 nm. Inset: a close-up scanning electron micrograph of the metasurface elements; scale bar: 1 μm. **c** Transmittance spectra of the metasurface measured using Fourier-transform infrared spectroscopy. Dashed line: central wavelength *λ*_L_ of the MIR fs pulses. Inset: simulated electric field **E** (arrows) and intensity |**E**|^2^ (color) inside a metasurface unit cell for the incident light at *λ* = *λ*_L_. The red region shows the Gaussian fit to the spectrum of the pump pulses. **d** Experimentally measured third harmonic generation (THG) in the metasurface (blue curve) and in an unstructured Si film (black curve) of the same thickness *h*. Both spectra are measured at the same MIR fluence of *F* = 2.3 mJ cm^−2^ (peak intensity *I* = 11 GW cm^−2^). A notable blue-shift of the upconversion peak from the metasurface with respect to the expected THG is a result of the plasma-induced acceleration of MIR photons. Inset: the same data plotted on the logarithmic scale. Gray region: the noise floor. The THG from the metasurface extends to *λ* = 1.06 μm, with intensities eight orders of magnitude higher than projected from the unstructured film (Gaussian fit given in dashed line): a clear indication of the emergence of the photons that are not present in the incident spectrum. **e** Spectra of MIR pulses transmitted through the metasurface for different input fluences revealing significant pulse self-modulation

Here, we design and experimentally realize a photon accelerating semiconductor infrared metasurface (PASIM) that undergoes rapid refractive index changes due to highly nonlinear photoinduced generation of free carriers (FCs) in silicon by a MIR pulse. The main idea of photon acceleration in a PASIM is given in Fig. 1a. Briefly, MIR photons interact with, and get trapped by, the metasurface. As FCs are generated by four-photon absorption (4PA), the resonant frequency of the metasurface blue-shifts, and the frequency of the trapped photons follows. Accelerated MIR photons then upconvert via the standard $\hat \chi ^{(3)}$ nonlinear process, resulting in the observed blue-shifting of the THG. The PASIM is designed to have a high-*Q* resonance at *λ*_R_ ≡ 2*πc*/*ω*_R_ = 3.62 μm that enables efficient four-photon FC generation at modest pulse intensities. This enables us to clearly observe the effect of PA on harmonics generation, with the peaks of the upconverted radiation appearing at frequencies of up to Ω_*n*_ ≈ 3.1*ω*_L_, where *ω*_L_ is the central frequency of the MIR pulses chosen at *ω*_L_ ≈ *ω*_R_ to maximize the effect. Moreover, we detect anomalous levels of nonlinearly generated signal with frequencies of up to ≈3.4*ω*_L_ in the wings of the THG spectra, corresponding to the spectral density enhancement of ≈10^8^ over the projected signal from an unstructured film. Such enhancement can only be explained by the frequency boost of the spectrally dense population of photons with initial frequencies around *ω*_L_, and their subsequent nonlinear conversion. An intuitive coupled-mode theory (CMT) model with time-dependent eigenfrequency *ω*_R_(*t*) and damping factor *γ*_R_(*t*) ≡ *ω*_R_(*t*)/2*Q*_R_(*t*) (where *Q*_R_(*t*) is a time-dependent quality factor of the metasurface) accurately captures most features of the experimental data and provides further insights into PA efficiency improvements, thus paving the way to future applications utilizing nonperturbative nonlinear nanophotonics.

## Results

### Metasurface design and fabrication

The metasurface was engineered to enhance the local fields, which is crucial for the efficiency of the nonlinear photon conversion and multiphoton absorption. In our design of the metasurface, we make use of high-*Q* collective resonances common in regular arrays of semiconductor particles^41,42,47^. The specific implementation of the PASIM comprised of nearly touching rectangular Si nanoantennas is shown in Fig. 1b. The quality factor of the resonance—and, hence, the local field enhancement—is controlled by the gap *g* between the rectangles (see Supplementary Note 1 for ultrahigh *Q*s of up to ≈10^4^). For photon acceleration of broadband femtosecond laser pulses, the *Q*-factor of the PASIM was designed to be moderate: *Q* ≈ 72, as experimentally confirmed by Fourier-transform infrared (FTIR) spectroscopy measurements using collimated beams^41^, as shown in Fig. 1c. Using full-wave simulations, we determine the local MIR field enhancement to be up to |**E**_loc_/**E**_0_|^2^ = 350, where **E**_0_ is the amplitude of the incident electric field polarized along the short dimension of the rectangle. The sharpness of the resonance is attributed to the very small net polarization current integrated over one unit cell^48^ as shown in the inset of Fig. 1c. The samples were fabricated according to a standard procedure described in Methods and Supplementary Note 2.

### Upconversion spectroscopy

Intense ultrashort MIR laser pulses with a variable nondestructive fluence in the 1 < *F* < 6 mJ cm^−2^ range, with central wavelength *λ*_L_ = 3.62 μm and time duration *τ*_L_ ≈ 200 ± 30 fs (spectral FWHM Δ*λ*_L_ ≈ 150 ± 50 nm) were focused onto the PASIM to 5 < *I* < 30 GW cm^−2^ peak intensity (see Supplementary Note 3 for the details of the optical setup). The advantage of operating the PASIM in the MIR regime is that the refractive index change scales as Δ*n*(*t*) ∝ − *N*_FC_(*t*)*λ*^2^, which will be crucial for PA; here, *N*_FC_(*t*) is the time-dependent FC density. Note that we neglect Kerr and thermal additions to the refractive index; corresponding discussion and estimates are provided in Supplementary Note 7. Kerr effect may play an important role in frequency conversion processes and may present an interesting avenue for further research in metasurfaces^49,50^.

The third-order nonlinear polarization, which is responsible for the third harmonic generation (THG), can be expressed in the perturbative regime as follows:

1 $${\mathbf{P}}^{(3)}(3\omega ) = \varepsilon _0\hat \chi ^{(3)}(3\omega = \omega + \omega + \omega ) \vdots {\mathbf{E}}(\omega ){\mathbf{E}}(\omega ){\mathbf{E}}(\omega ),$$

where $\hat \chi ^{(3)}(3\omega = \omega + \omega + \omega )$ is the third-order nonlinear susceptibility tensor of silicon, and **E**(*ω*) is the local electric field strength at the pump frequency *ω*. Although the resonant local field is confined to a very small volume of each nanoantenna, the cubic dependence of **P**^(3)^ on the local field enables considerable THG boost in the metasurface as compared to the unpatterned silicon film. This is indeed experimentally observed in the transmitted THG spectrum plotted in Fig. 1d for the metasurface (blue curve) versus the unpatterned film of the same thickness (black curve) cases: an order of magnitude spectrally integrated THG enhancement is provided by the metasurface, and a maximum conversion efficiency estimated at around 10^−9^. While similar magnitudes of THG enhancement and conversion efficiencies have been observed in the past^51^, much more revealing is the spectrum of the THG that has never been collected from rapidly changing metasurfaces, and which reveals several unambiguous signatures of the PA process.

We observe three features of the THG spectra that have not been previously experimentally observed or theoretically predicted for a solid-state medium. All three indicate that the THG takes place under nonperturbative conditions, when the optical properties of the metasurface are strongly modified while the laser pulse is interacting with it. First, the spectral peak is strongly blue-shifted with respect to its unperturbed 3*ω*_L_ (corresponding to $\lambda _{{\mathrm{TH}}}^{(0)} \approx 1.207\,{{\upmu {\mathrm m}}}$) spectral position measured with an unpatterned film, and with respect to the tripled frequency of the PASIM resonance at 3*ω*_R_. Second, because the spectral shift of the peak to ≈3.05*ω*_L_ is larger than the full widths at half maxima (FWHMs) of the unperturbed THG spectrum and of the metasurface resonance, there exists a high-frequency spectral region of the THG (*λ*_TH_ < 1.17 μm, on the blue side of the spectral peak) where the THG signal from the metasurface is more than two orders of magnitude higher than that from the Si film. This is a signature of the photons accelerated from the spectral peak of the incident pulse outside of its spectral width. In the inset of Fig. 1d, we show that signal with the wavelengths as short as *λ*_TH_ ~ 1.06 μm (i.e. more than 100 nm on the blue side of the highest energy signal detected from the unstructured film) can be detected. For example, for two specific wavelengths, *λ*_TH_ = 1.12 μm and *λ*_TH_ = 1.06 μm, the measured THG intensities from the metasurface are, respectively, four and eight orders of magnitude stronger than the corresponding projections calculated from the THG spectrum that was collected from the unpatterned film (dashed line). Previously, FC-induced blue-shifting of SHG was observed in metal-based metasurface^52,53^, but PA was weak due to the low *Q*-factor of the plasmonic resonance.

Finally, the THG spectrum reveals another counter-intuitive property of a PASIM: it is possible to resonantly enhance a nonlinear process (THG) without sacrificing the spectral bandwidth. All three features are related to the emergence of the new, higher energy photons, and can be attributed to photon acceleration due to the dynamic multiphoton FC generation. The transmitted MIR spectra shown in Fig. 1e reveal strong power dependence, thus providing further confirmation of the nonperturbative nature of the PA. No such dependence on the MIR fluence *F* was observed in bulk silicon (Supplementary Note 4), thus validating the key role of the resonantly excited hot spots that enable FC generation through 4PA.

**Fig. 2:**
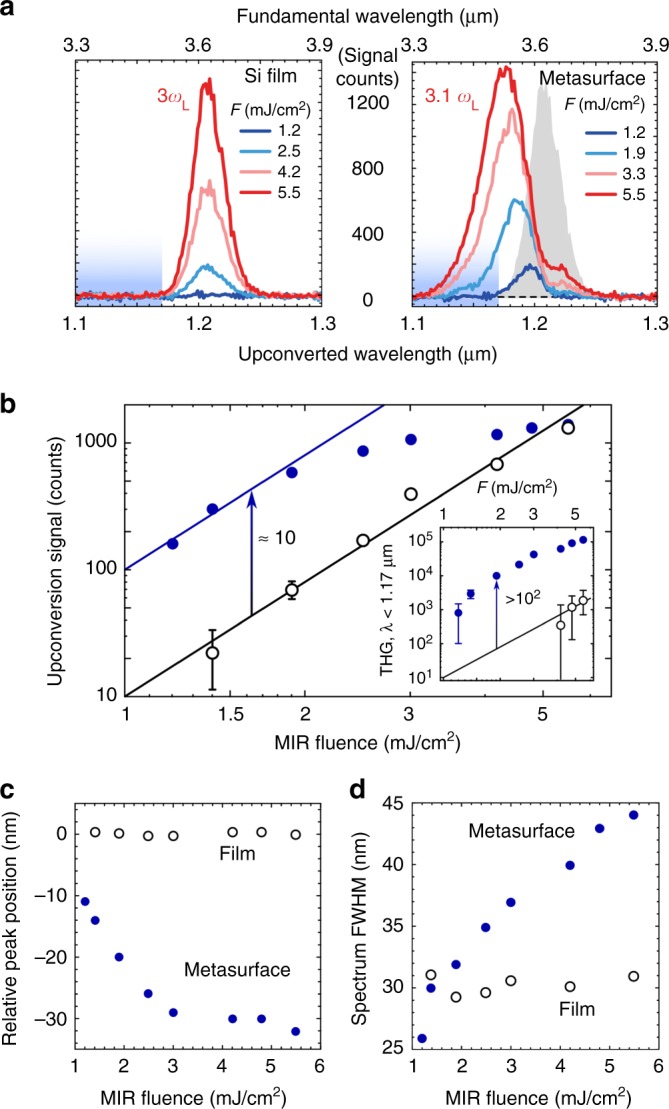
Spectral characteristics of blue-shifted harmonics in photon accelerating semiconductor infrared metasurfaces (PASIMs). **a** Third harmonic generation (THG) spectra measured for various input fluences for the unpatterned Si film and for the metasurface. Shaded gray area: THG from the unstructured film at the highest fluence *F*_max_ = 5.5 mJ cm^−2^. Shaded blue areas indicate the integration range of *λ*_TH_ < 1.17 μm. **b** Spectrally integrated third harmonic generation as a function of the mid-infrared fluence the film (open circles) and metasurface (closed circles), log–log scales. Lines denote the guide-to-the-eye *I*_TH_ ∝ *F*^3^ dependence. Inset: THG spectrum integrated over 1.1 μm < *λ*_TH_ < 1.17 μm as a function of *F* for the unstructured film (open circles) and the metasurface (filled circles). The THG spectrum from the PASIM reveals self-induced blue-shifting by ≈30 nm (**c**) and broadening by ≈50% (**d**) as a function of *F*; both effects are negligible for the unpatterned film. Error bars on the plots represent standard errors of the mean

To quantify the combined process that manifests as blue-shifted harmonics generation, we measured the NIR spectra as a function of the incident MIR fluence *F*, and compared them with the corresponding spectra generated in the unpatterned Si film (Fig. 2a). We observe that the spectral peak and width of the THG light generated in the PASIM can be controlled by incident fluence. The central THG wavelength can be blue-shifted by more than 30 nm, as shown in Fig. 2c, enabling the THG with center frequencies of up to ≈3.1*ω*_L_. In contrast with the common belief that the resonant enhancement of the THG must be accompanied by spectral narrowing, our results plotted in Fig. 2d indicate the opposite. The upconverted signal has a spectrum that is up to 50% broader than that from the unstructured film, which is a clear fingerprint of PA. We therefore conclude that the perturbative approach expressed by Eq. (1) fails, suggesting the need for a more accurate model of harmonic upconversion.

### Theoretical model

The observed blue-shifting, broadening and saturation of the THG spectra can be explained by a simple CMT involving a single cavity mode with a mode amplitude *p*(*t*), characterized by its time-dependent resonant frequency $\omega _{\mathrm{R}}(t) \equiv \omega _{\mathrm{R}}^0 + \Delta \omega _{\mathrm{R}}N(t)/N_{{\mathrm{max}}}$ and damping factor $\gamma _{\mathrm{R}}(t) \equiv \gamma _{\mathrm{R}}^0 + \Delta \gamma _{\mathrm{R}}N(t)/N_{{\mathrm{max}}}$, and coupled to the incident optical field $\tilde E(t)$ according to refs. ^54,55^:

2 $$\frac{{{\mathrm d}p(t)}}{{{\mathrm d}t}} + \left[ {i\omega _{\mathrm{R}}(t) + \gamma _{\mathrm{R}}(t)} \right]p(t) = \kappa \tilde E(t),$$

where *κ* is the coupling constant. Here, we assume a Gaussian incident laser pulse with $\tilde E(t) = \sqrt {\tilde I} \exp\left( { - i\omega _{\mathrm{L}}t - t^2/\tau _{\mathrm{L}}^2} \right)$, where *τ*_L_ = 105 fs and $\omega _{\mathrm{L}} = \omega _{\mathrm{R}}^0$, and $\tilde I$ is the intensity of the pump. The model does not aim to reproduce the peak intensity of the resulting harmonics, which is affected by the absolute normalization of the MIR pulse intensity. The resonant frequency/linewidth shifts are of greater importance for quantitative understanding of the PA’s role, and their estimation is described below.

**Fig. 3:**
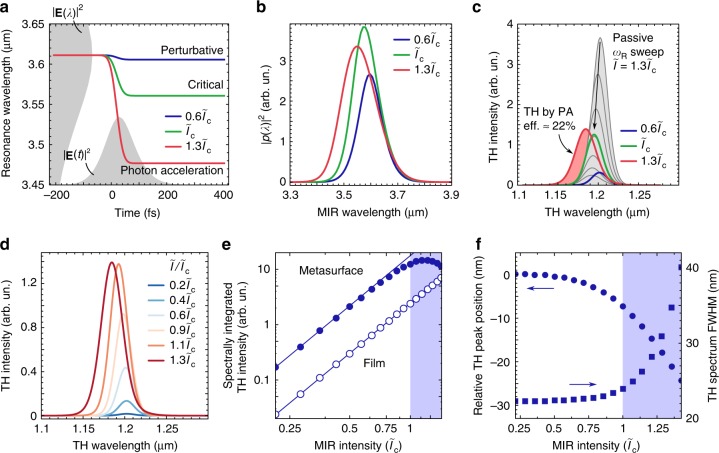
Theoretical model of the photon-acceleration-induced blue-shifted harmonics generation. **a** Time-dependent resonant frequency of the metasurface due to four-photon absorption free carrier generation plotted for three normalized peak intensities of the mid-infrared (MIR) pulse: $\tilde I = 0.6\tilde I_{\mathrm{c}}$ (perturbative regime, blue curve), $\tilde I = \tilde I_{\mathrm{c}}$ (critical regime, green curve), and $\tilde I = 1.3\tilde I_{\mathrm{c}}$ (nonperturbative photon acceleration regime, red curve). Shaded areas: the frequency-domain (on the left) and time-domain (on the bottom) profiles of the incident pulse. **b** Spectra of the MIR electric field inside the metasurface. **c**, **d** Predicted third harmonic generation (THG) spectra for different input MIR intensities. In (**c**), a comparison between the predictions of the models of time-dependent metasurface model (thick lines; coloring as in (**a**)) and of the time-independent (thin lines) metasurfaces. The family of time-independent metasurfaces satisfies $0 < \Delta \omega _{\mathrm{R}}/\omega _{\mathrm{R}}^0 < 4\%$ and $\tilde I = 1.3\tilde I_{\mathrm{c}}$. Filled red area: the portion of the spectrum inaccessible within the time-independent framework. **e** Spectrum-integrated THG intensity versus input intensity for the unpatterned film (open circles) and resonant metasurface (closed circles). THG from the film is simulated as from a significantly nonresonant structure with *γ*_R_ = 10^15^ s^−1^ with an intensity calibration coefficient that is adjusted for good agreement with the experiment at low intensities. Lines: guide-for-the-eye cubic dependences. **f** Intensity-dependent THG peak position (circles) and THG bandwidth (squares) generated by the metasurface. Shaded areas in (**e**) and (**f**): the nonperturbative photon acceleration regime

In Eq. (2), the unperturbed $\omega _{\mathrm{R}}^0$ and $\gamma _{\mathrm{R}}^0$ are obtained by fitting the transmission spectrum obtained with FTIR (see Fig. 1c), the coupling constant $\kappa = \sqrt {2\gamma _{\mathrm{R}}^0}$ ^54^ is calculated by neglecting nonradiative losses in the absence of FCs, and *N*_max_ is set to be the maximum FC density achieved in the experiments. The carrier-induced shifts are assumed to be proportional to carrier density *N*(*t*) < *N*_max_ produced via the 4PA:

3 $$N(t) = N_{{\mathrm{max}}}\frac{{2\sqrt 2 }}{{\tau _{\mathrm{L}}\tilde I_{{\mathrm{max}}}^4\sqrt \pi }}\mathop {\int}\nolimits_{ - \infty }^t {\left| {\tilde E(t\prime )} \right|^8} {\mathrm d}t\prime ,$$

where $\tilde I_{{\mathrm{max}}}$ is the maximum intensity. We further assume that *Δω*_R_ = 2×10^13^ s^−1^ and Δ*ω*_R_ = 2.5Δ*γ*_R_; these quantities are close to those obtained by pump–probe measurements (see Supplementary Note 5). The resulting dynamic frequency sweep profiles are shown in Fig. 3a for three characteristic intensities of $\tilde I = 0.6\tilde I_{\mathrm{c}}$, $\tilde I_{\mathrm{c}}$ and $1.3\tilde I_{\mathrm{c}}$, corresponding to three different regimes of PASIM operation; the meaning of the characteristic critical intensity $\tilde I_{\mathrm{c}}$ will be clarified below.

After numerically solving Eq. (2) to obtain the enhanced near-fields inside the metasurface ∝*p*(*t*), we calculate their spectra |*p*(*λ*)|^2^ (see Fig. 3b) and observe their significant blue-shifting and spectral broadening with the increasing incident intensity $\tilde I$. In the time domain, the latter feature translates into shorter and more intense bursts of the electric field in Si, thus giving rise to more intense nonlinear THG spectra *I*_TH_(*λ*_TH_) given by

4 $$I_{{\mathrm{TH}}}(\lambda _{{\mathrm{TH}}}) \propto \left| {\mathop {\int}\nolimits_{ - \infty }^\infty p^3(t)e^{i\omega _{{\mathrm{TH}}}t}{\mathrm d}t} \right|^2,$$

where *λ*_TH_ = 2*πc*/*ω*_TH_.

The resulting upconversion spectra are displayed in Fig. 3c, d for several values of the input light intensity $\tilde I$. In Fig. 3c, we illustrate the three regimes of PASIM operation by comparing the spectra with those obtained for a set of fixed *ω*_R_. At low intensities, e.g., $\tilde I = 0.6\tilde I_{\mathrm{c}}$ (blue curve), the shape of the upconverted spectrum does not differ from that produced by a time-independent metasurface with $\omega _{\mathrm{R}} = \omega _{\mathrm{R}}^0$. As the intensity increases, the upconverted light progressively blue-shifts, and for the critical intensity $\tilde I = \tilde I_{\mathrm{c}}$ (green curve) we start observing PA-induced photons that cannot be produced by any static metasurface with time-independent resonances. The transition to the PA regime is best illustrated for the intensity $\tilde I = 1.3\tilde I_{\mathrm{c}}$ (red curve in Fig. 3c). It is instructive to attempt reproducing the nonlinear spectrum by using a set of stationary resonant frequencies/linewidths corresponding to different values of *N*_*j*_/*N*_max_, where 1 ≤ *j* ≤ 6. We observe that, without invoking PA, it is impossible to reproduce the red curve in Fig. 3c by any one of such static metasurfaces, or even by using a weighted average of their corresponding THG spectra. The reason for that is the existence of highly blue-shifted THG photons (see the red shaded area).

By comparing the theoretical spectra shown in Fig. 3d with their experimental counterparts shown in Fig. 2a, we find that the simple model of PA described by Eqs. (1–4) semi-quantitatively captures the key spectral features of the PASIM-based THG: simultaneous increase in intensity, blue-shift, and spectral width with increasing laser intensity. The model rather accurately captures the saturation of the THG as shown in Fig. 3e, the value of the blue-shift (~25 nm), and the spectral broadening from the initial linewidth of the resonance (~22 nm) up to ~40 nm, as shown in Fig. 3f. Even though the dynamic resonance sweep is a very complex process, especially because of the highly localized nature of FC generation, we have demonstrated that our model can explain the main features of the experiment, and can be potentially used for optimizing future metasurface designs.

## Discussion

We have estimated the energy portion of the upconverted radiation that is unreachable by a passive frequency sweep, *η*_PA_ ≈ 22%, by calculating the shaded area in Fig. 3c relative to the area covered by the gray curves. Comparing to previous demonstrations of PA in microcavities^20,21^, which yielded up to *η*_PA_ ≈ 50%, we note that the value of *η*_PA_ demonstrated here is achieved at much larger relative wavelength shifts (about 2.7% of the central wavelength versus <0.5% shown before), and without any external pump. Moreover, the ultrathin nature of the PASIM potentially enables tunable high-harmonics generation despite their finite absorption in Si. While comparable PA-associated blue-shifts of the THG have been observed^17^ in ionizable gases, the required laser intensities were in the PW cm^−2^ range, i.e., almost five orders of magnitude higher than those used in our experiments.

**Fig. 4:**
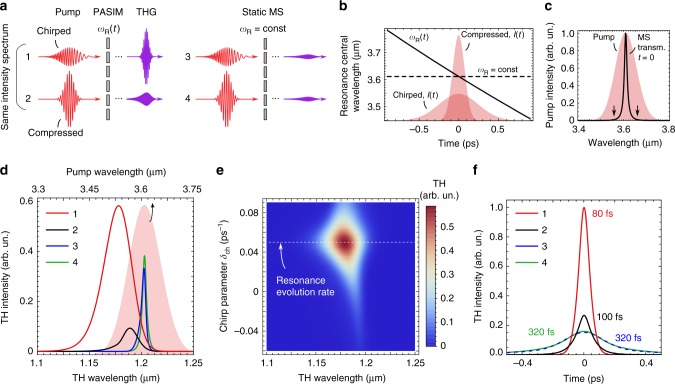
Nonlinear interaction of broadband pulses with narrow-band metasurfaces. **a** Third harmonic generation (THG) from a narrow-band photon accelerating semiconductor infrared metasurface (PASIM) (cases 1 and 2) and static metasurface (MS) (cases 3 and 4) using broadband pulses, chirped or transform-limited (compressed). **b** Evolution of the MS resonance frequency *ω*_R_(*t*) for a PASIM (solid line) and a static MS (dashed line) superimposed with the temporal profiles (shaded areas) of the chirped (*τ*_L_ = 500 fs) and compressed (*τ*_L_ = 150 fs) mid-infrared (MIR) pulses with the same energy and spectral width. **c** Intensity spectrum of the pump (shaded area) and transmittance spectrum of the metasurfaces at *t* = 0 (black curve): Δ*ω*_L_ ≈ 8Δ*ω*_R_. The arrows indicate the sweep range of the PASIM’s *ω*_R_(*t*) within the temporal full width at half-maximum of the chirped MIR pulse. **d** Spectra of THG generated in cases 1–4. Shaded area referenced to the top axis: the spectrum of the MIR pulses. **e** THG emission from the PASIM for different values of the chirp parameter *δ*_ch_. Both the bandwidth and efficiency peak at *δ*_ch_ = *α*. **f** THG pulses generated in cases 1–4 after perfect compression, illustrating the possibility of generating ultrashort (*τ*_TH_ < 80 fs) bursts of the THG radiation by the PASIM. Metasurfaces’ parameters: *α* = 0.05 ps^−1^ (for PASIM), Δ*ω*_R_ = 1 ps^−1^ (for both metasurfaces)

As an application example, we will discuss how the PA mechanism enables a new approach to improving photons’ capture and nonlinear conversion: utilizing optical pulse shaping/chirping^56,57^ to engineer the right- and left-hand sides of Eq. (2). We demonstrate that significant enhancement of the bandwidth and intensity of the THG can be achieved by matching the instantaneous frequency of a chirped laser pulse to the time-dependent resonant frequency of the metasurface. Figure 4 shows the calculated THG spectra produced by the interaction of a time-dependent PASIM with *ω*_R_(*t*) = *ω*_R_(0)(1 + *αt*) (where *α* = 0.05 ps^−1^) with an incident chirped laser pulse whose electric field is given by

5 $$E_{{\mathrm{in}}}(t) = E_0e^{ - t^2/\tau _{\mathrm{L}}^2} \times e^{ - i\omega _{\mathrm{L}}t} \times e^{ - i\delta _{{\mathrm{ch}}}\omega _{\mathrm{L}}t^2/2},$$

where the last term describes a linear frequency chirp with the normalized rate of change *δ*_ch_. By choosing the FWHM of the pulses’ intensity spectrum Δ*ω*_L_, and the instantaneous width of the metasurfaces’ resonance Δ*ω*_R_ in such a way that ${\mathrm{\Delta }}\omega _{\mathrm{L}} \gg {\mathrm{\Delta }}\omega _{\mathrm{R}}$, we demonstrate that the benefits of the high-*Q* metasurface and a broadband incident laser pulse can be combined to achieve broadband THG with high conversion efficiency. Four cases are considered in Fig. 4a: cases 1–2 of a PASIM with a linearly evolving resonance frequency *ω*_R_(*t*) = *ω*_R_(0)(1 + *αt*) excited by either a chirped or a transform-limited (compressed) MIR pulse, and cases 3–4 of a static metasurface with *ω*_R_(*t*) = *ω*_R_(0) excited by the same pulses. The corresponding parameters of the pulses and metasurface are listed in the caption of Fig. 4. The chirped and compressed pulses are chosen to have identical spectral intensities.

The predictions of the CMT calculations for these four cases are plotted in Fig. 4d. The PASIM excited by both the chirped pulse (case 1) and the compressed pulse (case 2) produces broadband THG, with former being much more efficient than the latter. The nonlinear response of the PASIM strongly depends on *δ*_ch_, including its sign. Specifically, the enhancement by the PASIM is the highest if the chirp parameter *δ*_ch_ matches the evolution rate *α* of the PASIM’s resonance. This can be clearly observed in Fig. 4e, where the THG spectra from the PASIM are plotted for different values of *δ*_ch_. To our knowledge, the PASIM concept is the first proposal for a chirp-sensitive nonlinear metasurface. In contrast, the passive metasurface (cases 3 and 4) produces narrow-band THG with low efficiency because they do not utilize the portion of the optical bandwidth that is outside of the metasurface bandwidth.

The importance of utilizing the entire bandwidth of the laser pulse can be most easily appreciated from the following observation. We predict that a compressed THG signal from a PASIM pumped by an optimally chirped laser pulse can be as short as *τ*_TH_ ≈ 80 fs despite the long lifetime of the mode *τ*_R_ ≡ 1/*γ*_R_ ≈ 1 ps. This is accomplished by using an additional passive dispersive element (e.g., a pair of compressing gratings) that turns a long chirped THG pulse emerging from the PASIM into a transform-limited short pulse while preserving its bandwidth. The comparison between the four cases discussed above is shown in Fig. 4f. It is apparent that only in case 1 an intense short THG pulse is produced. These results establish that a high-*Q* photon-accelerating metasurface can exhibit simultaneously efficient and broadband response, and thus provides a path toward surpassing the time-bandwidth limit found in systems with resonances^58^.

As an outlook, here we speculate on the possibilities of scaling our approach to other wavelength ranges, as well as outline several potential applications of PASIMs. Many applications benefit from efficient frequency conversion in the near-infrared and the visible. In order to scale the design down by a factor of 6, so as to push the PASIM operation down the visible, the smallest feature (the gap size) should be on the order of about 35 nm at the same height-to-gap aspect ratio of 2:1. Current e-beam resist technology can produce sub-20-nm resist feature sizes^59^ that, in appropriate etching conditions, will result in the desired pattern. As far as the specific semiconductors, GaP is arguably the best candidate for applications of PA in the visible, for it has a large refractive index (*n* = 3.2–4) and band gap (2.25 eV) required for resonant operation using collective modes used in this work. The main obstacle to large frequency shifts at shorter wavelengths is the *λ*^2^-scaling of the Drude term. However, this is not the only known term that affects the refractive index of semiconductors. In our previous work^39^, GaAs was used near the band gap, where the refractive index was affected by band filling and band shrinkage effects^60^. Therefore, by judicious choice of materials and band gap engineering in ternary semiconductors, PA may be extended to near-IR and visible frequencies.

Below we briefly discuss a potential application of PASIMs: filling the spectral gap between high optical harmonics, with the objective of generating satellite-free isolated attosecond pulses—something that is currently accomplished using bulky optical components^61^. We propose to utilize the process of high-harmonic generation (HHG) by an MIR pulse which nonlinearly interacts and gets spectrally broadened by a time-dependent PASIM. The role of the PASIM is to sufficiently broaden the spectrum of the pulse so that adjacent (*N*′th and *N* + 1′th, where $N \gg 1$) harmonics spectrally overlap. A similar PASIM based on a high-mobility semiconductor (GaAs) with *Q* ~ 10^3^ would be utilized, and its resonance wavelength swept by Δ*λ*_R_/*λ*_R_ ~ 0.1, where *λ*_L_ ≈ *λ*_R_ = 3.6 μm and $\tau _{\mathrm{L}}^{{\mathrm{comp}}}\sim 170\,{\mathrm {fs}}$ are the wavelength and FWHM duration of the transform-limited intense MIR pulse used for metasurface-enhanced HHG. Such sweep of the metasurface resonance frequency requires FC concentrations on the order of *N*_FC_ ~ 3×10^18^ cm^−3^ due to the low effective mass of electrons in GaAs^60^. In Supplementary Fig. 8, we show that the spectrum of the *N* = 15th harmonic generated from a PASIM is sufficiently broad to fill the spectral gap between the *N*th and *N* + 1st harmonics. Because the PASIM-induced spectral broadening of the *N*th harmonic is proportional to *N*, and high harmonics up to *N* = 32 have already been produced in solids^62^, we anticipate that PASIMs could play a pivotal role in obtaining resonant solid-state HHG continua for the generation of attosecond pulses in the extreme UV.

To summarize, we have provided the first demonstration of photon acceleration in ultrathin semiconductor metasurfaces by observing a blue-shifted THG with central frequencies of up to 3.1*ω*_L_. Relative wavelength shifts as high as 2.7% have been observed under moderate laser intensities owing to excitation of collective high-*Q* metasurface resonances. Using a CMT with time-dependent mode parameters, we have validated our experimental findings and estimated the overall photon acceleration efficiency at around *η*_PA_ ≈ 22%. In the measured spectra, new frequencies of up to ≈3.4*ω*_L_ have emerged, with the spectral intensity of up to eight orders of magnitude higher than the projected intensity from an unstructured silicon film. These findings indicate that photon-accelerating nanostructures represent a novel time-dependent nonlinear photonic platform that can find various applications in novel pulsed light sources.

## Methods

### Sample fabrication and characterization

Samples of silicon metasurfaces were fabricated at the Cornell Nanoscale Facility (CNF) from a silicon-on-insulator wafer (600 nm undoped Si device layer on top of a 460 μm top-grade sapphire from University Wafer) using the following recipe. The substrate was cleaned with acetone, isopropanol and O_2_ plasma; PMMA 495 was spun to form a 400-nm-thick layer and baked for 15 min at 170 °C; PMMA 950k was spun to form a 100-nm-thick layer and baked for 15 min at 170 °C; E-spacer conducting layer was spun at 6000 rpm; the pattern was exposed at 1000 μC cm^−2^ (JEOL 9500FS) and developed in MIBK:IPA 1:3 solution; a 60-nm-thick Cr mask was electron-beam-evaporated and lifted off in sonicated acetone for 1 min; the pattern was transferred to the silicon layer through HBr reactive ion etch (Oxford Cobra). Finally, Cr was removed with the commercially available Cr wet etchant.

### Infrared spectroscopy

Bruker Vertex 80 FTIR spectrometer was upgraded to an external transmittance spectroscopy setup as described elsewhere^41^ that enables collimated MIR spectroscopy, with the MIR beam focused to a spot size of about 300 μm in diameter using a pinhole imaging technique. The transmitted beam was sent to the detector and Fourier-analyzed by the spectrometer. Normalization was done using the signal from the clear sapphire area.

### Nonlinear optical measurements

In Supplementary Fig. 3, a schematic of the optical setup used for nonlinear measurements is shown. The Extreme Mid-IR (EMIR) optical parametric amplifier (OPA) is a homebuilt KNbO_3_/KTA 3-crystal/3-pass OPA. EMIR is pumped by The Ohio State University’s GRAY laser, a homebuilt 80-fs Ti:Sapphire chirped pulse amplification system with a central wavelength of 780 nm and 4 mJ per pulse. The repetition rate of EMIR can be varied nearly continuously between 1 and 500 Hz using an external Pockels-cell-based pulse picker. EMIR was used to generate 200-fs mid-IR pulses with up to 40 μJ per pulse. The output wavelength of EMIR can be varied continuously from *λ* = 2.7 to 4.5 μm. For the experiments, the MIR (idler) beam was fixed at *λ* = 3.62 μm. The 780 nm NIR, MIR, and *λ* = 1 μm (signal) output beams are separated spatially, with the 1-μm signal being dumped and 780 nm pump being retained for use in pump–probe experiments. The residual NIR and MIR beams are roughly collimated to a size of about 2.5 mm. The NIR pulses were found to have a pulse duration of 200 ± 15 fs.

Output modes were characterized for several different wavelengths using a WinCamD-FIR2-16-HR 2 to 16 μm Beam Profiler System. Residual NIR pulse length was characterized using a BBO crystal-based near-IR autocorrelator. MIR pulse duration was measured using an AGS-crystal-based MIR autocorrelator for 3 and 3.6 μm.

MIR spectra were obtained using a homebuilt spectrometer based on a ThorLabs GR1325-30035 blazed ruled diffraction grating with a blaze wavelength of 3.5 μm as the dispersion element and the beam profiler sensor as the detector array. On the setup schematic, an inset demonstrates a typical image of the diffracted MIR beam. The spectrometer was calibrated with an A.P.E. Wavescan USB MIR spectrometer, which, due to a low sensitivity and operation speed, could not be used for the routine MIR spectroscopy.

For pump–probe and upconversion spectroscopy, the horizontally polarized MIR pulses first pass through a waveplate-polarizer assembly for precise energy control. The pulses travel through a variable delay line after which they are recombined with the NIR pulses via a dichroic mirror. The NIR pulses follow a separate but similar path. The collinear beams are focused using a CaF_2_ *f* = 100 mm plano-convex lens. In the sample plane, the spot sizes were found to be 300 μm FWHM for MIR and 400 μm FWHM for NIR. Both spots fit within the 500 × 500 μm^2^ structured area of the metasurfaces. The relative delay between MIR and NIR pulses was controlled dynamically using either the manual MIR delay line or the electronically controlled NIR delay line with sub-ps resolution. For self-tuning of the resonance, the NIR beam is blocked with a beam block. MIR fluences were varied from 1 to 6 mJ cm^−2^ (see Supplementary Table 1) and NIR fluences were varied from <1 to 4 mJ cm^−2^ for the experiments. As a control method, an Si wafer was pumped in place of the sample. With NIR beam blocked, contamination of scattered light from the NIR and 1 μm signal was measured at the sample location. The NIR content was found to be 0.5 pJ per pulse and 1 μm signal was estimated to be of order 1 pJ per pulse. These pulse energies were determined to be insignificant to affect the sample during the experiment.

Upon transmission through the sample, the MIR beam and any upconversion signal were collected with a CaF_2_ *f* = 50 mm bi-convex lens. Any residual NIR was filtered using an Si window. In one configuration, a blazed grating/MIR camera combination is used as a high-resolution MIR spectrometer. In another configuration, a commercial Ocean Optics NirQuest spectrometer (900–2500 nm) is used for detection of the upconverted radiation. THG signal was power-calibrated using the signal beam from the OPA at *λ* = 1.2 μm that had a known power, after being attenuated by a set of neutral density filters with a known (measured) transmittance at this wavelength. By dividing the mean power of the THG beam by the mean power pump beam, an estimate maximum conversion efficiency of 10^−9^ was obtained.

### Finite element simulations

We used COMSOL Multiphysics to model the response of the metasurfaces (no free carriers) by defining the computational domain as a slab with the dimensions of *p*_*x*_ × *p*_*y*_ × 3 μm, where *p*_*x*_ = 2.1 μm and *p*_*y*_ = 2 μm. Periodic boundary conditions were used for the domain boundaries parallel to the *x*−*z* and *y*−*z* planes, and perfectly matched layers were used for the domain boundaries parallel to the *x*−*y*. The dimensions of the metasurface were chosen to match those obtained from the SEM image. Wavelength-independent refractive indices of *n*_Si_ = 3.45 and $n_{{\mathrm{Al}}_2{\mathrm{O}}_3} = 1.7$ were used for Si and sapphire, respectively.

## Supplementary information

Supplementary Information

## Data Availability

The data that support the findings of this study are available from the corresponding author upon reasonable request.
