# Supplementary material for: Photon acceleration and tunable broadband harmonics generation in nonlinear time-dependent metasurfaces
Source: Nat Commun. 2019 Mar 22;10:1345. doi: 10.1038/s41467-019-09313-8 (PMC6430811; doi:10.1038/s41467-019-09313-8)
Supplement: Supplementary file 1 — Supplementary Information [file 41467_2019_9313_MOESM1_ESM.pdf]

Photon acceleration and tunable broadband harmonics generation in nonlinear time-dependent metasurfaces. Supplementary information

Maxim R. Shcherbakov, *et al.*

(Dated: February 27, 2019)

Supplementary Note 1

High-Q Metasurface Design

The main considerations for the design of the photon accelerating semiconductor infrared metasurfaces (PASIMs) were outlined in similar realisations of high-Q semiconductor nanoparticle arrays [1,2]. The basic physics behind crafting a high-quality-factor metasurface is to create a planar waveguide system with a route to couple light in and out by periodic corrugations. A very simple system we will consider here consists of conventional rectangular silicon waveguides with periodic through-notches that match the momentum of the initially normally incident light to a waveguide mode: $2\pi/p_y = \beta(\omega)$, where p_y is the periodicity of corrugations and $\beta(\omega)$ is the propagation constant of the waveguide mode; see the structure of the metasurface in Supplementary Figure 1a. As a result, for the frequencies that satisfy the equation above, strong transmission dips are observed, as shown in Supplementary Figure 1b for a metasurface comprised of domino-shaped dielectric resonator antennas (DRAs). The geometry parameters of the metasurface, defined in the Supplementary Figure 1a, are as follows: $w_x = 0.84$ µm, $w_y = 1.82$ µm, $p_x = 2$ µm, $p_y = 2.1$ µm, and $h = 600$ nm. By changing the gap width g, one can tune the coupling between the DRAs, thereby affecting the Q-factor of the resonance. This effect is shown in Supplementary Figure 1c, where the Q factor of the metasurface is plotted as a function of the gap size.

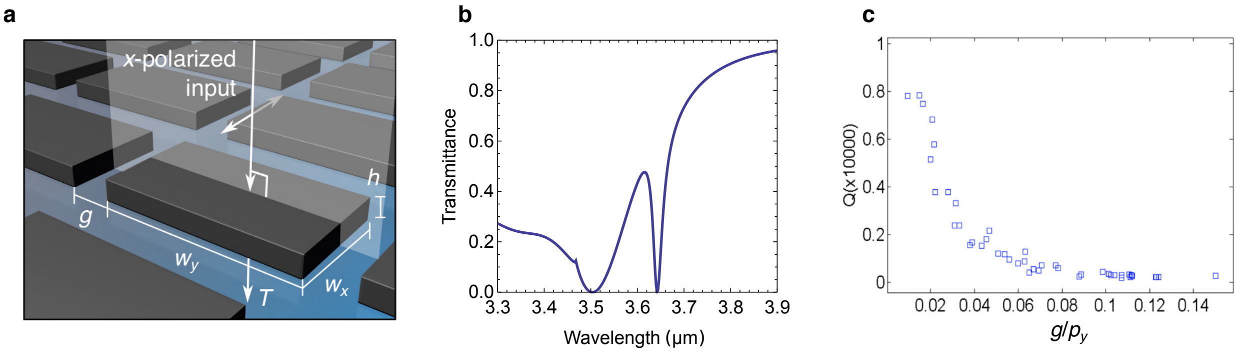

Supplementary Figure 1. **High-Q metasurfaces design.** **a**, The geometry of the PASIM with the dimensions and illumination parameters outlined. **b**, The calculated transmittance spectra of the PASIM used in the experiments. A sharp transmittance dip is seen at the coupling wavelength around $\lambda = 3.64$ µm. **c**, Q-factor of the resonance dip as a function of the relative gap width g/p_y. For $p_y = 2.1$ µm and $g = 20$ nm, the Q-factor can reach $Q \approx 8 \times 10^3$.

Supplementary Note 2

Sample Fabrication

The PASIM fabrication procedure is illustrated in Supplementary Figure 2(a). We used a silicon-on-insulator wafer (600 nm undoped Si device layer on top of a 460 μm top-grade sapphire from University Wafer) and the following recipe. The substrate was cleaned with acetone, isopropanol and O_2 plasma; PMMA 495 was spun to form a 400-nm-thick layer and baked for 15 min at 170°C; PMMA 950k was spun to form a 100-nm-thick layer and baked for 15 min at 170°C; E-spacer conducting layer was spun at 6000 rpm; the pattern was exposed at 1000 μC cm^{-2} (JEOL 9500FS) and developed in MIBK:IPA 1:3 solution; a 60-nm-thick Cr mask was electron-beam-evaporated and lifted off in sonicated acetone for 1 minute; the pattern was transferred to the silicon layer through HBr reactive ion etch (Oxford Cobra). Finally, Cr was removed with the commercially available Cr wet etchant. In Supplementary Figure 2(b), the choice of the sapphire substrate is justified by showing high transparency for mid-infrared radiation with $\lambda \approx 3.6$ μm.

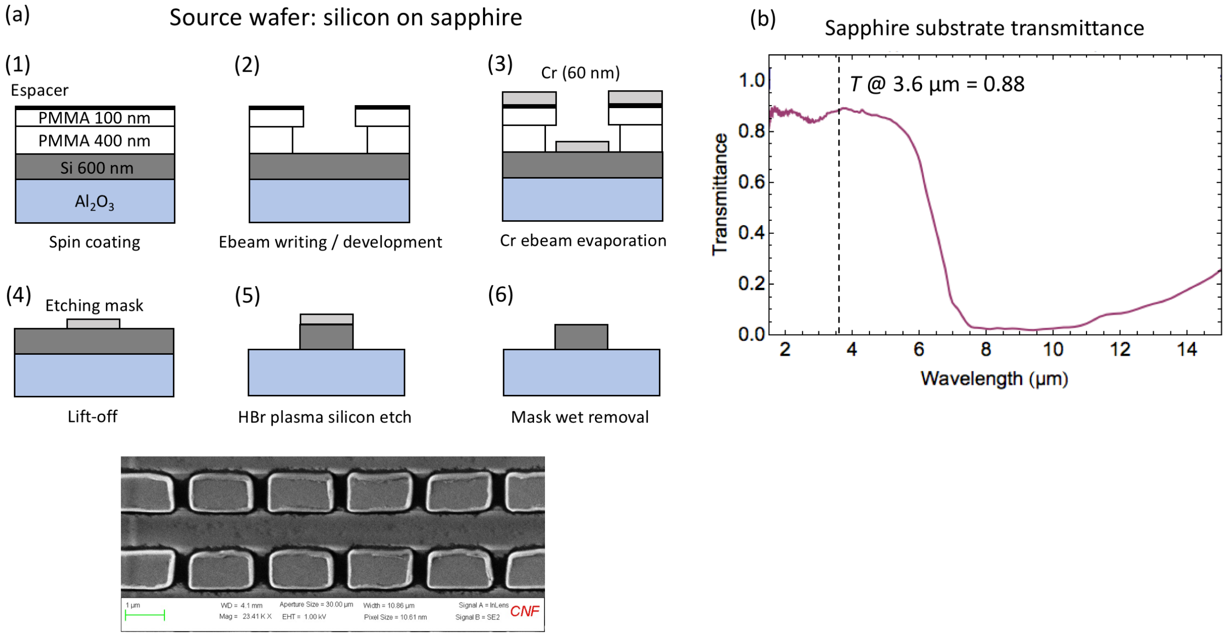

Supplementary Figure 2. **Sample fabrication and characterisation.** (a) Sample fabrication process steps (1-6) and a scanning electron microscope image of the sample. (b) Transmittance of the sapphire substrate (purple curve) showing large transmittance in the spectral region around 3.6 μm.

Supplementary Note 3

Nonlinear-Optical Measurements

In Supplementary Figure 3, a schematic of the optical setup used for nonlinear measurements is shown. The Extreme Mid-IR (EMIR) optical parametric amplifier (OPA) is a homebuilt $KNbO_3$/KTA 3-crystal/3-pass OPA. EMIR is pumped by The Ohio State University's GRAY laser, a homebuilt 80-fs Ti:Sapphire chirped pulse amplification system with a central wavelength of 780 nm and 4 mJ per pulse. The repetition rate of EMIR can be varied nearly continuously between 1 and 500 Hz using an external Pockels-cell-based pulse picker. EMIR was used to generate 200-fs mid-IR pulses with up to 40 μJ per pulse. The output wavelength of EMIR can be varied continuously from $\lambda = 2.7$ to 4.5 μm. For the experiments, the MIR (idler) beam was fixed at $\lambda = 3.62$ μm. The 780 nm NIR, MIR, and $\lambda = 1$ μm (signal) output beams are separated spatially, with the 1-μm signal being dumped and 780 nm pump being retained for use in pump–probe experiments. The residual NIR and MIR beams are roughly collimated to a size of about 2.5 mm. The NIR pulses were found to have a pulse duration of 200 ± 15 fs.

Output modes were characterized for several different wavelengths using a WinCamD-FIR2-16-HR 2 to 16 μm Beam Profiler System. Residual NIR pulse length was characterized using a BBO crystal based near-IR autocorrelator. MIR pulse duration was measured using an AGS-crystal-based MIR autocorrelator for 3 and 3.6 μm.

MIR spectra were obtained using a home-built spectrometer based on a ThorLabs GR1325-30035 blazed ruled diffraction grating with a blaze wavelength of 3.5 μm as the dispersion element and the beam profiler sensor as the detector array. On the setup schematic, an inset demonstrates a typical image of the diffracted MIR beam. The spectrometer was calibrated with an A.P.E. Wavescan USB MIR spectrometer, which, due to a low sensitivity and operation speed, could not be used for the routine MIR spectroscopy.

For pump–probe and upconversion spectroscopy, the horizontally polarized MIR pulses first pass through a waveplate-polarizer assembly for precise energy control. The pulses travel through a variable delay line after which they are recombined with the NIR pulses via a dichroic mirror. The NIR pulses follow a separate but similar path. The collinear beams are focused using a CaF_2 $f = 100$ mm plano-convex lens. In the sample plane, the spot sizes were found to be 300 μm FWHM for MIR and 400 μm FWHM for NIR. Both spots fit within the 500×500 μm^2 structured area of the metasurfaces. The relative delay between

Supplementary Table 1. Parameters of the mid-infrared beam: incident intensity I_{inc}, incident fluence F_{inc}, estimated equivalent intensity in the metasurface hotspot I_{hs}.

	I_{inc} (GW cm^{-2})	F_{inc} (mJ cm^{-2})	I_{hs} (TW cm^{-2})
Minimum	5	1	< 1.75
Maximum	30	6	< 10.5

MIR and NIR pulses was controlled dynamically using either the manual MIR delay line or the electronically controlled NIR delay line with sub-ps resolution. For self-tuning of the resonance, the NIR beam is blocked with a beam block. MIR fluences were varied from 1 to 6 mJ cm^{-2} (see Supplementary Table 1) and NIR fluences were varied from < 1 to 4 mJ cm^{-2} for the experiments. As a control method, a Si wafer was pumped in place of the sample. With NIR beam blocked, contamination of scattered light from the NIR and 1 μm signal was measured at the sample location. The NIR content was found to be 0.5 pJ per pulse and 1 μm signal was estimated to be of order 1 pJ per pulse. These pulse energies were determined to be insignificant to affect the sample during the experiment.

Upon transmission through the sample, the MIR beam and any upconversion signal were collected with a CaF_2 $f = 50$ mm bi-convex lens. Any residual NIR was filtered using a Si window. In one configuration, a blazed grating/MIR camera combination is used as a high resolution MIR spectrometer. In another configuration, a commercial Ocean Optics NirQuest spectrometer (900–2500 nm) is used for detection of the upconverted radiation. THG signal was power-calibrated using the signal beam from the OPA at $\lambda = 1.2$ μm that had a known power, after being attenuated by a set of neutral density filters with a known (measured) transmittance at this wavelength. By dividing the mean power of the THG beam by the mean power pump beam, an estimate maximum conversion efficiency of 10^{-9} was obtained.

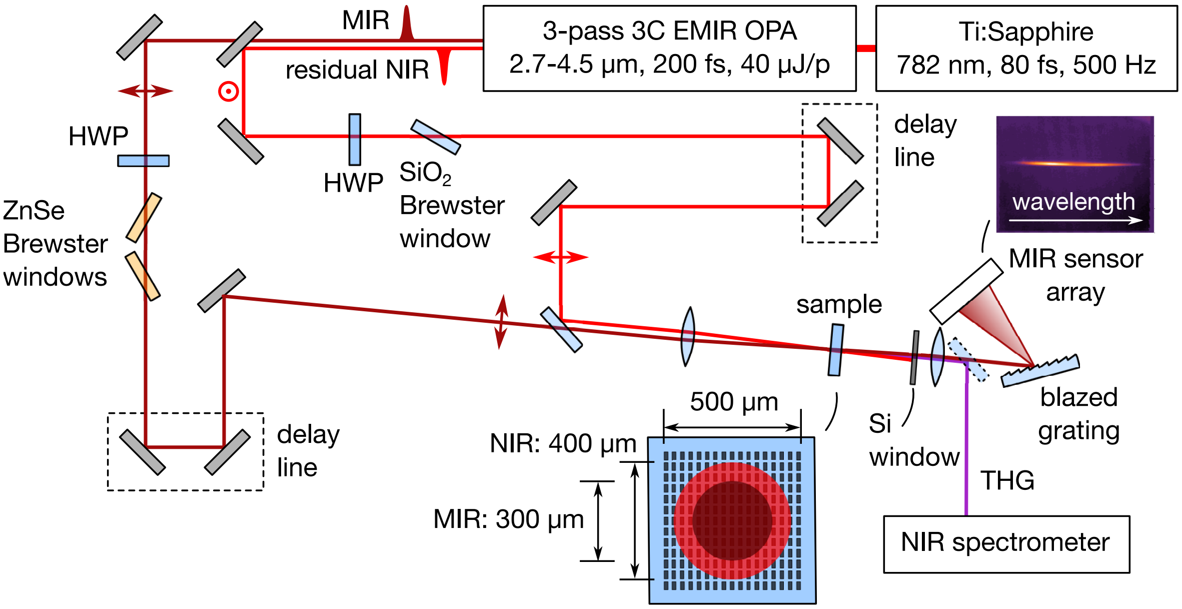

Supplementary Figure 3. **Nonlinear measurements setup.** Optical setup is based on a Ti:Sapphire amplifier system coupled to an three-crystal (3C) extreme mid-infrared (EMIR) optical parametric amplifier (OPA) capable of producing femtosecond laser pulses with the carrier wavelength tunable from 2.7 to 4.5 μm. Half-wave plates (HWP) are used for power management of both the near-infrared (NIR) pump beam, outlined with light red, and the mid-infrared (MIR) probe beam, outlined with dark red. THG is the third harmonic generation beam.

Supplementary Note 4

Power-Dependent Transmittance of the PASIM Enabled by Four-Photon Absorption

The nonlinear properties of the PASIM can be interpreted only after the physics behind the resonance modification by mid-IR pulses is fully understood. This is facilitated by measuring the transmitted pulse spectra as a function of the incident pulse fluence (see Supplementary Figure 4) which controls the FC generation. Note that undoped silicon has negligible linear absorbance [3] in the studied spectral range because the bandgap of silicon $E_g = 1.12$ eV is more than three times larger than the MIR pulse carrier energy of $\hbar\omega = 0.35$ eV. Therefore, the interaction of MIR pulses with the metasurfaces is only affected by high-order processes, such as the four-photon absorption (4PA). We make estimates of the FC concentration via:

$$N = 2F(1 - e^{-\alpha h})/4hE_{\text{pump}}, \tag{1}$$

where F is the incident pulse fluence, α is the 4PA-induced absorption constant, h is the thickness of the medium, and E_{pump} is the energy of a pump photon; the factor of 2 stands for a pair of generated FCs and the factor of 4 stands for the four photons needed for an absorption act. The 4PA-induced absorption is calculated with $\alpha = \beta_4 I^3$, where $\beta_4 = 3.5 \cdot 10^{-4}$ cm^5/GW^3 is the 4PA coefficient [4] and I is the input intensity. At an intensity of $I = 10$ GW/cm^2 (fluence $F \approx 2$ mJ/cm^2), in a bulk silicon wafer, $\alpha = 3.5$ cm^{-1}, and the FC density is only $N \approx 3 \cdot 10^{16}$ cm^{-3}. Not surprisingly, essentially no self-modulation was observed for the 600 μm-thick silicon wafer, as shown in Supplementary Figure 4a for four fluence values.

However, the hot spots inside the metasurface (Fig.1a,b of the main text) enable a much higher FC density. With the local intensity of $I_{\text{local}} = 3.5$ TW/cm^2 (due to the 350-fold intensity enhancement shown in Fig. 1b of the main text), the 4PA-induced absorption constant approaches $\alpha \approx 10^{10}$ cm^{-1}, which can induce full absorption of the incident pulse by the hot spots. Of course, Supplementary Eq.(1) fails under high intensities due to pump depletion; however, these estimates suggest that giant self-modulations of MIR pulses are

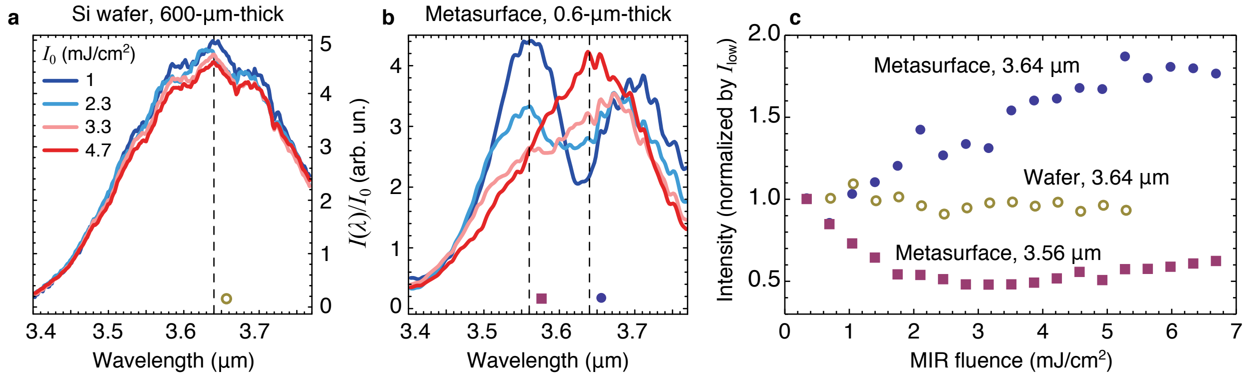

Supplementary Figure 4. **Power-dependent transmittance of the mid-infrared beam.** **a**, Spectra of mid-infrared (MIR) pulses transmitted through a 500-μm-thick silicon wafer, divided by the input pulse fluence. **b**, Spectra of MIR pulses transmitted through the metasurface, divided by the input pulse fluence. The same set of pump fluences is used as in panel **a**. A dip that starts off $\lambda = 3.64$ μm corresponds to the high-Q resonance observed in the FTIR transmittance spectrum in Fig. 1b of the main text. **c**, A comparison of the fluence-dependent transmittance on the resonance ($\lambda = 3.64$ μm, closed circles) and off the resonance ($\lambda = 3.56$ μm, closed squares) to that of the wafer at $\lambda = 3.64$ μm (open circles).

possible with our metasurfaces. Such fluence-dependent modifications of the resonance frequency and lifetime are shown in Supplementary Figure 4b. At low fluences (blue curve), the transmitted pulse contains a dip at $\lambda_{\text{dip}} = 3.64$ μm $\approx \lambda_R$, which is in good agreement with the FTIR measurements. At higher fluences, the dip experiences considerable modifications, including blue-shift and broadening, and at the maximum fluence of $F = 5.5$ mJ cm^{-2}, the dip is seen no more, as it has moved out of the spectral bandwidth of the mid-IR pulses.

Additionally, even at the lowest fluence, there is clear evidence of the PA. Specifically, the spectral component of the transmitted pulse are clearly redistributed from the dip region to a new global peak at $\lambda_{\text{peak}} = 3.56$ μm which is absent in the linear transmittance spectrum shown in Fig. 1b of the main text. This suggests that a significant fraction of the photons from the most populated portion of the original spectrum around $\lambda = \lambda_L$ are trapped by the metasurface resonance, and then accelerated to shorter wavelengths by the resonance blue-shifting during the FC generation. For this process to be efficient, femtosecond control of the FC generation is crucial. Specifically, it is important that significant FC generation takes place around the peak of the laser intensity. If it starts too early in the pulse, then by the arrival time $t_{\max}$ of the intensity maximum of the MIR pulse, the metasurface resonance frequency $\omega_R(t = t_{\max})$ will have shifted too far to the blue (where the number of the incident photons is small), and will have decreased its quality factor $Q_R(t = t_{\max})$ to the value that is insufficient for photon trapping. The temporal dynamics of the resonant frequency $\omega_R(t)$ is illustrated in Fig.3a of the main text.

Supplementary Note 5

Plasma-Induced Blue-Shift and Damping of the Metasurface Resonance: Pump-Probe Experiments

We can appreciate the values for the FC-induced metasurface resonance modification by measuring the MIR pulse transmittance spectra upon FC injection by an external NIR pump. The spectra of the pulses transmitted through the sample were measured at different pump powers and/or pump–probe delays. Here we will concentrate on the cases of the pump preceding the probe by $\Delta\tau = 1$ ps to ensure that the probe interacts with a non-evolving metasurface whose resonance has been blue-shifted by the earlier pump pulse. Such choice of the pulse delays ensures that no photon acceleration takes place. To obtain the transmittance spectra, The acquired spectra were divided by the initial spectrum of the pulses transmitted through the substrate in the absence of the pump pulses . In order to extract the position of the resonance, we fitted the transmittance spectra near the dips by a parabolic dependence, which is the second-order Taylor expansion of the Lorentzian line shape. Several example of the curves after the division, along with the fit-extracted dip positions, is shown in Supplementary Figure 5. The maximum wavelength shift was observed to be $\Delta\lambda = 140$ nm (approximately a 4% frequency shift), or $\Delta\omega_R = 2\pi c\lambda^{-2}\Delta\lambda \approx 1.8 \cdot 10^{13}$ s^{-1}. The resonance damping factor was calculated from the manually defined FWHM of the resonance. Having taken into account the unperturbed (radiative) value of $\gamma_R^0 = 7 \cdot 10^{12}$ s^{-1}, the peak non-radiative increment to the resonance damping is estimated as $\Delta\gamma_R \approx 7.5 \cdot 10^{12}$ s^{-1}. In the modeling, to reasonably reproduce the experimental results, we found it necessary to use the following values: $\Delta\omega_R = 2 \cdot 10^{13}$ s^{-1} and $\Delta\gamma_R = 5 \cdot 10^{12}$ s^{-1}, which are fairly close to those obtained from the pump–probe measurements.

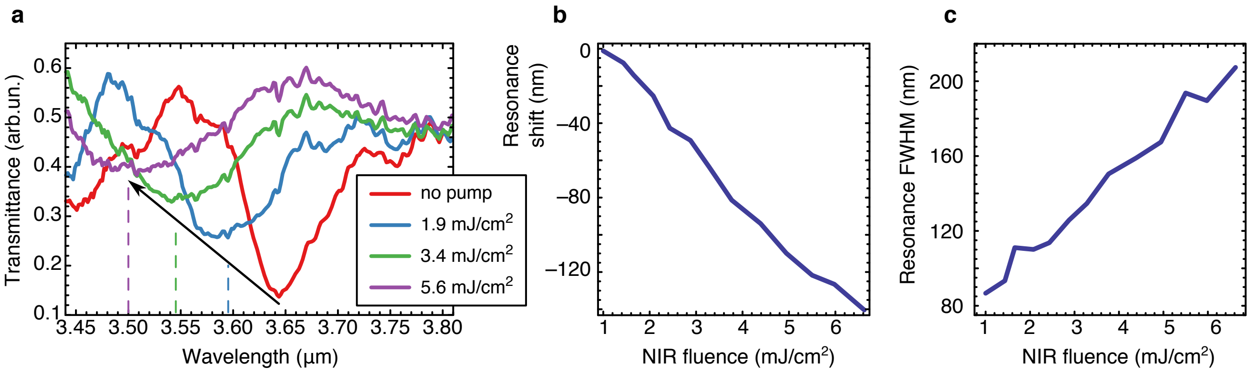

Supplementary Figure 5. **Pump-induced tuning of the metasurface resonance. a**, Pump-induced transmittance changes in the metasurfaces for several near-IR pump fluences color-coded according to the legend. **b**, Retrieved resonance shift as a function of the near-IR beam fluence. **c**, Retrieved resonance FWHM as a function of the near-IR beam fluence.

Supplementary Note 6

Blue-shifted Harmonics from an Off-resonant PASIM

In addition to the experiments performed on the metasurface (referred to as the MS1) with an unperturbed resonant wavelength $\lambda_R^{(1)}$ coincident with the central wavelength of the MIR pulse $\lambda_L = 3.62$ µm ($\lambda_R^{(1)} = \lambda_L$), we have also performed similar measurements on a second metasurface (referred to as the MS2). MS2 is designed to have a shorter unperturbed resonant wavelength $\lambda_R^{(2)} = 3.56$ µm, i.e. even in the absence of FC generation the MS2 is blue-shifted with respect to λ_L. The measured linear transmittance through MS2 is shown in Supplementary Figure 6a. The power-dependent spectra of the non-integer THG for the MS2 are shown in the Supplementary Figure 6b. We observe that the peak of the THG spectrum is blue-shifted as the MIR fluence increases. Similar behavior was observed for the MS1 as well. There are, however, three key differences in the THG spectra collected from MS2 and MS1.

First, we observe that the MS2 produces two spectral peaks (unlike the single peak for the MS1, see Fig.2c of the main manuscript). Because the long-wavelength peak is close to $\lambda_L/3$, we associate it with a non-resonant contribution to the THG from the detuned sample. Similarly to the unstructured film case, its position stays within a narrow ≈ 2 nm range for all fluences. This peak is produced by the frequency trippling of the photons with the highest spectral density centered at λ_L. These photons do not undergo photon acceleration

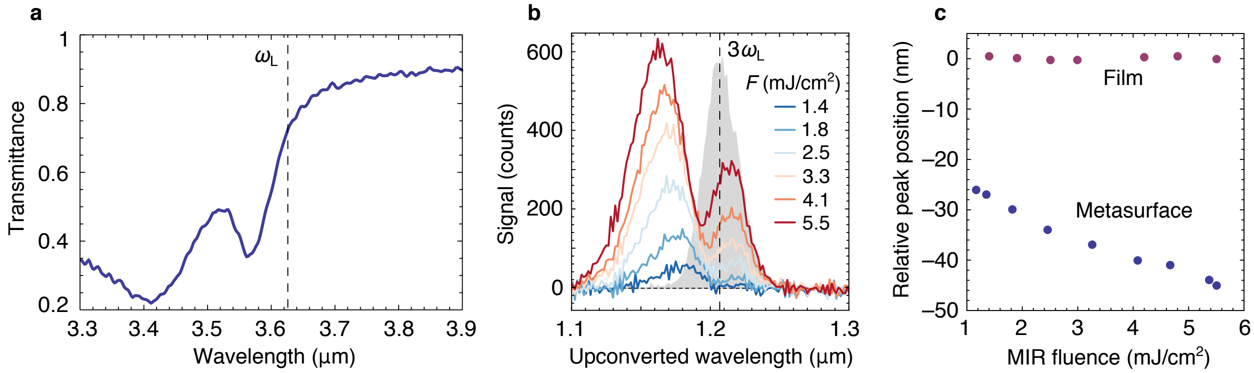

Supplementary Figure 6. **Blue-shifted harmonics from an off-resonant metasurface** Linear and nonlinear optical responses of the low-Q, off-resonant metasurface sample. **a**, Linear transmittance spectrum of the metasurface. The dip at $\lambda_R^{(2)} = 3.56$ μm indicates the excitation of a resonant collective mode of the metasurface. **b**, The spectra of the frequency-tripled signal as a function of the mid-infrared (MIR) fluence for the metasurface. The grey shaded area is the third harmonic spectrum from an unstructured silicon film pumped at a fluence of 5.5 mJ cm^{-2}. **c**, The position of the main upconversion peak in panel **b** as a function of the MIR fluence (blue dots). The same dependence measured for an unstructured Si film is shown with purple dots.

because they are not trapped by the metasurface. The main (short-wavelength) spectral peak is analogous to the blue-shifted peak observed in the experiments with the MS1. It is produced by the photons in the wings of the original MIR spectrum, whose wavelength coincides with $\lambda_R^{(2)}$ and is consequently shortened via photon acceleration. Therefore, the response of the MS2 combines the features of the resonant response of the MS1 (acceleration and frequency tripling of the trapped photons), and of the non-resonant response of the unpatterned Si film (non-resonant frequency tripling without photon acceleration).

Second, the peak spectral intensity of the THG from the MS2 corresponding to the highest MIR fluence is smaller by a factor 2 that the corresponding peak intensity from the MS1. This reduction of the nonlinear signal is due to two factors: (i) the lower Q-factor translates into lower field enhancement, and (ii) there are fewer photons that are captured and accelerated by the evolving metasurface because of the initial blue shift of the MS2's resonance with respect to the spectral density peak of the incident MIR pulse.

Finally, the blue-shifting of the central position of the THG peak does not appear to saturate even at the highest MIR fluence. as a function of fluence. This indicates that fewer

FCs are produced inside the hot spots of the MS2 at a given fluence when compared with the FCs' density in the MS1. Therefore, the distortion of the spatial mode's profile remains small for the MS2, even at the highest MIR fluence.

Supplementary Note 7

On Kerr and Thermo-optic Effects

In a semiconductor subjected to an intense laser pulse, the main contributions to the refractive index modulation are photogenerated free carriers, Kerr effect and thermo-optic effect. In this work, we assume that the main contribution to the refractive index is the one from the photogenerated free carriers. There are two main reasons why Kerr and thermo-optic effects can be assumed smaller than that from free carriers.

First, in silicon, both Kerr and thermo-optic additions to the refractive index are positive: $\Delta n_{\mathrm{Kerr}} = n_2 I$, where $n_2 \approx 3 \times 10^{-14}$ cm^2/W [4], $\Delta n_{\mathrm{therm}} = T dn/dT$, where $dn/dT = 2 \times 10^{-4}$ 1/K [5]. Positive n_2 and dn/dT mean the anticipated resonance shifts are to the red part of the spectrum; instead, only blue shifts are observed.

Second, thermal effects are known to affect the optical properties of materials after the system has thermalized, which usually happens several picoseconds after the pulse arrives. Since we are using a low-repetition-rate source, the system cools down before the next pulse comes, ensuring that thermo-optic effects do not affect our measurements.

Interestingly though, the Kerr effect can be important at the mode hotspots, as estimated from the known values of n_2 for Si. We arrive at $\Delta n_{\mathrm{Kerr}} \approx 0.1$ at an input intensity of 10 GW/cm^2, if the local-field-induced equivalent intensity in the hot spot of the metasurface is 350 times that of the input field. This is on the same order with the estimated $\Delta n_{\mathrm{FC}} \approx -0.1$ we experimentally observe at this intensity. However, due to the observed blue-shifts, and potential FC-induced damping of the hotspot intensity, we believe the FC contribution dominates the response of the metasurface. Nevertheless, we predict that Kerr nonlinearities may start playing an important role under different, yet realistic experimental conditions, such as longer wavelengths and lower intensities.

Supplementary Note 8

Derivation of Eq.(3)

Eq.(3) of the main text is a result of a well-established rate equation for four-photo-

absorption-induced FC generation (see, for instance, Eq.(1) from [6]):

$$\frac{dN}{dt} = KI^4(t), \tag{2}$$

where K is a coefficient proportional to the 4PA constant. Then, we integrate it assuming the input pulse is Gaussian:

$$N(t) = K \int_{-\infty}^{t} I^4(t')dt', \tag{3}$$

where $I = |E_{\text{in}}(t)|^2 = \tilde{I} \exp(-2t^2/\tau_L^2)$. Finally, to find K, we use:

$$N(+\infty) = N_{\text{max}} \left(\frac{\tilde{I}}{\tilde{I}_{\text{max}}} \right)^4 . \tag{4}$$

This equation ensures (a) that the FC density after the pulse is gone is proportional to $\tilde{I}^4$, and (b) that at $\tilde{I} = \tilde{I}_{\text{max}}$, $N(+\infty) = N_{\text{max}}$. From:

$$N(t) = K \int_{-\infty}^{\infty} I^4(t')dt' = K\tilde{I}^4 \sqrt{\frac{\pi \tau_L}{8}}, \tag{5}$$

applying Eqs.(S4,S5) to Eq.(S3), one comes up with Eq.(3) of the main text.

Supplementary Note 9

Reproduction of Fig.1d by CMT

Here, we used the same set of parameters we used to obtain Fig.3 of the main text, with the exception that the transmitted fundamental pulse spectrum is calculated instead of the THG. Output fields are calculated via $E_{\text{out}}(t) = E_{\text{in}} - \kappa p(t)$ where the coupling constant $\kappa = \sqrt{2\gamma} = 3.7$ ps$^{-1/2}$, then the quantity $E_{\text{out}}(t)$ is Fourier-transformed to $E_{\text{out}}(\omega)$, and $|E_{\text{out}}(\omega)|^2$ plotted for four different intensities in Suppl. Fig. 7. We find similar behavior in our theory as in the measured curves: for the lowest intensity, there is a dip in the center of the initially-Gaussian pulse spectrum; as intensity increases, the dip blue-shifts and broadens; at the maximum intensity, the dip is broadened and blue-shifted considerably away from its initial position.

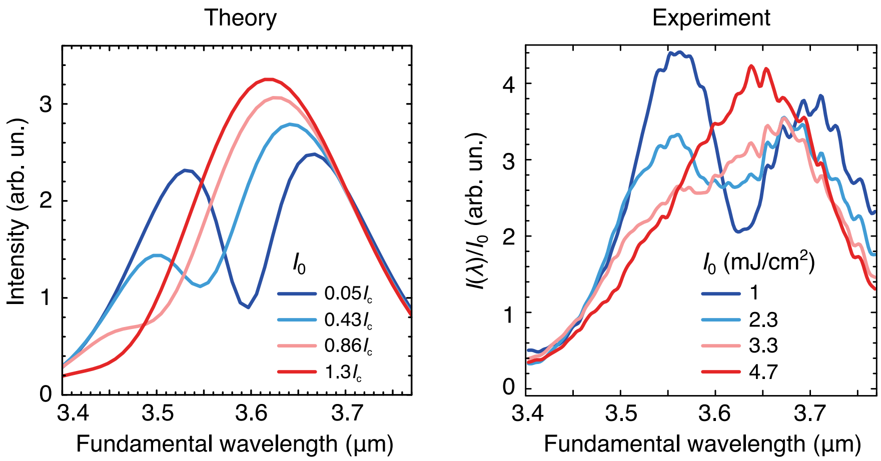

Supplementary Figure 7. **Coupled mode theory (CMT) results at the fundamental frequency.** Left: CMT results for pulse transmitted light spectra as a function of incident power. The parameters of the model are the same as those in Fig.3 of the main text, except for here, the transmitted fields are given instead of THG, as well as the out-coupling constant is let to be a free parameter. Right: corresponding experimental results, Fig.1d of the main text.

Supplementary Note 10

Broadband High Harmonic Generation

The ability of PASIMs to manipulate the bandwidth of the pulsed radiation and its optical harmonics can be applied for generation of high harmonic continuum and for spectral manipulation.

Specifically, here, we theorize an experimentally realistic setting where PASIMs show broadband generation of high optical harmonics enough to spectrally overlap the neighboring orders and produce broadband radiation in the UV. We consider a metasurface based on a high-mobility semiconductor (GaAs) with a resonance Q-factor of 10^3. The central wavelength of the resonance is being swept by 0.1 within the fwhm duration of the pulse around a value of 3.2 μm, while the (chirped) pulse duration is assumed to be 170 fs. Such a sweep is predicted to be possible at maximum FC plasma concentrations of around $N_{\mathrm{FC}} \approx 3 \cdot 10^{18}$ cm^{-3} [7]. Note that at such FC densities, FC absorption can start playing detrimental role on the quality factor of the resonance. Although at the chosen wavelength, the absorption constant is on the order of < 100 cm^{-1} ($n'' < 0.003$), which we think is insufficient to significantly impede the operation of the cavity, further studies are needed to pinpoint to role of FC absorption and its mitigation.

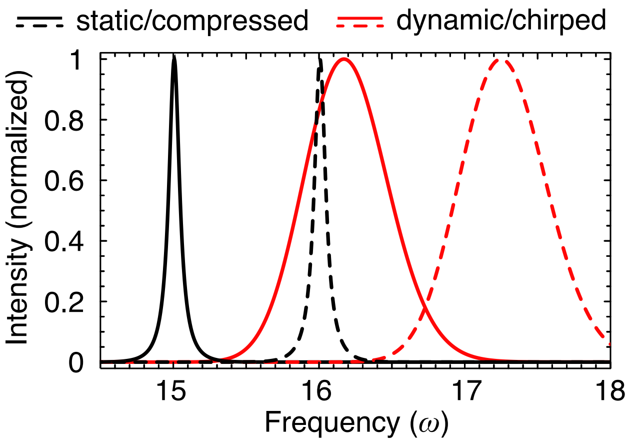

Supplementary Figure 8. **Merging high harmonics from a photon accelerating semiconductor infrared metasurface (PASIM).** Spectra of the 15th (solid lines) and 16th (dashed lines) optical harmonics generated by a static metasurface and a compressed femtosecond pulse (black) and a PASIM and a properly chirped pulse (red), revealing the spectral overlap of the accelerated harmonics.

In Supplementary Figure 8 we show the spectra of the 15th and 16th harmonics emitted from the time-varying metasurface pumped by the chirped pulse: the photon-accelerated emission spectrum consists of the harmonic continuum. Since the amount of blue-shift in the Nth harmonic grows as a power of N, and HHG of up to $N = 32$ has been observed in solids [8], we find this result to be an important step toward HHG UV continuum generation, and one can expect metasurface cavities to play a pivotal role in attosecond metrology in the extreme UV.

Supplementary Note 11

Role of $\gamma\tau$ parameter and high Q-factors in efficient PA

We start with a single-mode CMT:

$$\frac{da(t)}{t} + [i\omega(t) + \gamma]a(t) = \sqrt{\gamma}s(t), \tag{6}$$

where $a(t)$ is the mode amplitude, $\omega(t)$ is its eigenfrequency that we assume time-dependent, γ is its damping constant that we assume fixed, and $s(t)$ is the excitation field. We assume our system has only one mode, which is a good approximation for multimode systems that evolve slowly so as to be orthogonal at any given time. The exact solution of the CMT

equation is:

$$a(t) = \sqrt{\gamma} \int_{-\infty}^{t} dt' e^{-\gamma(t-t') - i[\phi(t) - \phi(t')]} s(t'), \tag{7}$$

where $\phi(t) = \int_{-\infty}^{t} \omega(\xi) d\xi$ is the phase advance of the mode, and the initial condition $a(-\infty) = 0$ was used. Typically, the excitation will come in a form of a pulse:

$$s(t) = A(t) e^{-i\psi(t)}. \tag{8}$$

Here, $A(t)$ is a real, slowly varying envelope function that satisfies $\lim_{t \to \pm\infty} A(t) = 0$, and $\psi(t)$ is the phase of the excitation. For a given $\omega(t)$ there exists an optimal evolution of the excitation phase:

$$\psi(t) = \int_{-\infty}^{t} \omega(\xi) d\xi + i\varphi \tag{9}$$

Under the optimal excitation phase, the solution takes the form of:

$$a(t) = \sqrt{\gamma} e^{-\gamma t - i\phi(t)} \int_{-\infty}^{t} dt' e^{\gamma t'} A(t'), \tag{10}$$

Let us assume that the output radiation is defined as $s_+(t) = \sqrt{\gamma} a(t)$. If the lifetime of the cavity is small with respect to the pulse duration τ, or $\gamma\tau \gg 1$, from Eq.(S10), we can assume $e^{\gamma t'}$ to be a slowly varying function with respect to $A(t)$, so that:

$$s_+(t) \approx A(t) e^{-i\psi(t)} = s(t), \tag{11}$$

and no frequency conversion takes place. It means that the most interesting regime for frequency synthesis is being $\gamma\tau$ on the order of or smaller than 1, meaning high-Q resonators produce more PA photons than the low-Q ones.

To illustrate the importance of a high-Q metasurface for producing measurable photon acceleration, we have carried out numerical simulations of the Eq.(2) of our manuscript for two cases of time-varying resonators: (i) a high-Q resonators ($Q = 70$), and (ii) a low-Q resonator ($Q = 7$). In both cases, a Gaussian transform-limited laser with the central wavelength of $\lambda_L = 3.61$ μm and duration $\tau_L = 200$ fs was used as an input. These two cases, respectively, correspond to the resonator bandwidth being within the laser bandwidth (the PA case described in our manuscript), and the opposite case of the laser bandwidth being entirely within the resonator bandwidth (the case described in Refs.[52, 53] of the

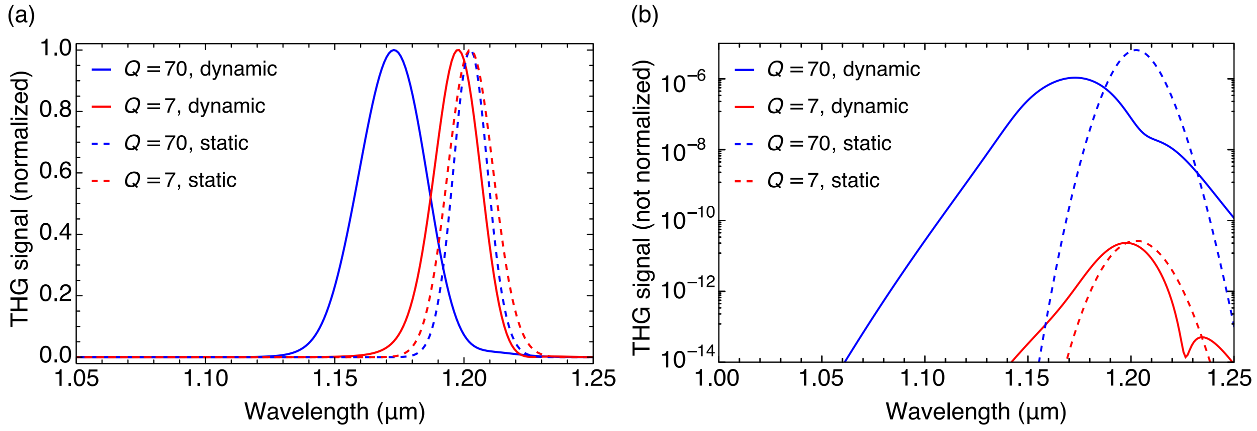

Supplementary Figure 9. **The role of the Q-factor in the photon acceleration process.** THG interaction of an input MIR pulse with high- and low-Q resonators that are either time-dependent (dynamic) or time-independent (static). MIR pulse parameters: $\lambda_L = 3.61$ μm, $\tau_L = 200$ fs. The resonant frequencies of the dynamic resonators are swept from $\lambda_R(-\infty) = 3.61$ μm to $\lambda_R(+\infty) = 3.44$ μm. The largest spectral broadening and blue-shifting of the THG pulse is obtained using a high-Q dynamic resonator (solid blue line). Clearly, the blue-shift and the spectral broadening achieved using a high-Q metasurface are much more prominent than those in the case of a low-Q metasurface. (a) Normalized THG signal in the linear scale, (b) THG signal before normalization in the logarithmic scale.

main text). Two time protocols for varying the resonant wavelengths of the metasurface were chosen: (a) static metasurface, and (b) a metasurface with a resonant wavelength swept from $\lambda_R(-\infty) = 3.61$ μm to $\lambda_R(+\infty) = 3.44$ μm. Therefore, both the broadband and narrow-band time-varying metasurfaces are swept over an identical wavelength range that exceeds the linewidth of the incident MIR laser pulse.

In Supplementary Figure 9, the normalized spectra of the near-IR pulses produced via THG mechanism are plotted for all four cases. The low-Q metasurface, which is used here to emulate the broad-band resonator of Refs.[52,53] of the main text, produces a spectrally-reshaped laser pulse (red solid line) that appears to be slightly on the blue side of the THG pulse produced from a static resonator (red dashed line). The spectral shift of order is almost an order of magnitude smaller than the spectral shift of the resonant frequency. The strong spectral overlap between the THG spectra from the static (red dashed line) and dynamic (red solid line) spectra is a clear proof that photon acceleration can be neglected in

the case of a broadband (low-Q) resonator. In stark contrast, the high-Q resonator, which is used here to emulate the narrow-band metasurfaces used in our experiments, produces a broadband and strongly blue-shifted optical pulse (solid blue line). A significant fraction of frequency-tripled photons exist in the part of the spectrum (e.g., $\lambda_{THG} < 1.15$ μm) where no progenitor photons ($\lambda_{THG} < 3.45$ μm) exist in the original input pulse. Therefore, no amount of spectral filtering by a resonator with a blue-shifted resonance frequency could have given rise to the THG photons. Additionally, we find that narrow-band (high-Q) resonators produce nonlinear responses that are several orders of magnitude stronger than their broadband (low-Q) counterparts. Unlike the normalized spectra plotted Supplementary Figure 9(a), the absolute magnitudes of the THG signals are plotted in Supplementary Figure 9(b) for the two resonators. The advantage of using temporally changing high-Q resonators is obvious: the resulting THG signals are not only spectrally broader than their low-Q counterparts, but also 6 orders of magnitude stronger.

SUPPLEMENTARY REFERENCES

[1] Yang *et al.*, Nat. Commun. **5**, 5753 (2014).

[2] Parry *et al.*, Appl. Phys. Lett. **111**, 053102 (2017).

[3] Soref *et al.*, J. Opt. A **8**, 840 (2006).

[4] Gai *et al.*, Laser Photon. Rev. **7**, 1054 (2013).

[5] Komma *et al.*, Appl. Phys. Lett. **101**, 041905 (2012).

[6] Hasselbeck *et al.*, J. Opt. Soc. Am. B **14**, 1616 (1997).

[7] Osamura and Murakami, Jap. J. Appl. Phys. **11**, 365 (1972).

[8] You *et al.*, Nat. Commun. **8**, 724 (2017).

[9] Haus, *Waves And Fields In Optoelectronics* (Prentice-Hall, 1984).

[10] Fan et al., J. Opt. Soc. Am. A **20**, 569 (2003).
